# Psychologist in a Pocket: Lexicon Development and Content Validation of a Mobile-Based App for Depression Screening

**DOI:** 10.2196/mhealth.5284

**Published:** 2016-07-20

**Authors:** Paula Glenda Ferrer Cheng, Roann Munoz Ramos, Jó Ágila Bitsch, Stephan Michael Jonas, Tim Ix, Portia Lynn Quetulio See, Klaus Wehrle

**Affiliations:** ^1^ The Graduate School University of Santo Tomas Manila Philippines; ^2^ Department of Medical Informatics RWTH University Hospital Aachen Germany; ^3^ Communication and Distributed Systems RWTH Aachen University Aachen Germany; ^4^ College of Science Department of Psychology University of Santo Tomas Manila Philippines

**Keywords:** depression, Psychologist in a Pocket, lexicon development, text analysis

## Abstract

**Background:**

Language reflects the state of one’s mental health and personal characteristics. It also reveals preoccupations with a particular schema, thus possibly providing insights into psychological conditions. Using text or lexical analysis in exploring depression, negative schemas and self-focusing tendencies may be depicted. As mobile technology has become highly integrated in daily routine, mobile devices have the capacity for ecological momentary assessment (EMA), specifically the experience sampling method (ESM), where behavior is captured in real-time or closer in time to experience in one’s natural environment. Extending mobile technology to psychological health could augment initial clinical assessment, particularly of mood disturbances, such as depression and analyze daily activities, such as language use in communication. Here, we present the process of lexicon generation and development and the initial validation of Psychologist in a Pocket (PiaP), a mobile app designed to screen signs of depression through text analysis.

**Objective:**

The main objectives of the study are (1) to generate and develop a depressive lexicon that can be used for screening text-input in mobile apps to be used in the PiaP; and (2) to conduct content validation as initial validation.

**Methods:**

The first phase of our research focused on lexicon development. Words related to depression and its symptoms based on the Diagnostic and Statistical Manual of Mental Disorders, Fifth Edition (DSM-5) and in the ICD-10 Classification of Mental and Behavioural Disorders: Clinical Descriptions and Diagnostic Guidelines classification systems were gathered from focus group discussions with Filipino college students, interviews with mental health professionals, and the review of established scales for depression and other related constructs.

**Results:**

The lexicon development phase yielded a database consisting of 13 categories based on the criteria depressive symptoms in the DSM-5 and ICD-10. For the draft of the depression lexicon for PiaP, we were able to gather 1762 main keywords and 9655 derivatives of main keywords. In addition, we compiled 823,869 spelling variations. Keywords included negatively-valenced words like “sad”, “unworthy”, or “tired” which are almost always accompanied by personal pronouns, such as “I”, “I’m” or “my” and in Filipino, “ako” or “ko”. For the content validation, only keywords with CVR equal to or more than 0.75 were included in the depression lexicon test-run version. The mean of all CVRs yielded a high overall CVI of 0.90. A total of 1498 main keywords, 8911 derivatives of main keywords, and 783,140 spelling variations, with a total of 793, 553 keywords now comprise the test-run version.

**Conclusions:**

The generation of the depression lexicon is relatively exhaustive. The breadth of keywords used in text analysis incorporates the characteristic expressions of depression and its related constructs by a particular culture and age group. A content-validated mobile health app, PiaP may help augment a more effective and early detection of depressive symptoms.

## Introduction

Depression has been identified as the most prevalent clinical disorder and main cause of illness among college students and young adults [1,2]. In this age group, depression manifests itself in poor academic performance, problems in communication, strained relations with family and peers, lesser interaction time with friends, and increased frequency in drinking and smoking behaviors [3]. It may also be signaled by loss of weight or increase in appetite and poor sleeping patterns [4]. In worse cases, severe depression is manifested in suicidal thoughts, plans and attempts, and self-harming behaviors [5].

Timely recognition of depressive symptoms is not without challenges. First, mood is transient, changing, and must be captured at the time of experience [6]. Second, primary strategies or traditional techniques used in assessment, such as face-to-face interviews and questionnaires, may overlook the presence of depression or produce negative recall bias [7]. Third, cultures influence the understanding and acceptance of mental illness. In many Asian countries, stigma, fears of misconception, and “losing one’s face” [8-10] hinder help-seeking behaviors. In the Philippines, despite having the highest depression incidence (over 4.5 million) in Southeast Asia, only few will seek help [11], while a significant number underreport self-harm and suicide cases due to religious reasons [12]. Others remain ill-informed, avoid acknowledging their condition, or treat depression as normal sadness. Filipino college students who experience depression tend to suffer in silence, although symptoms are highly observed [3].

Language, and the words that one uses, is a reflection of an individual’s mental state, exposing how one organizes and interprets his/her world and revealing thoughts and feelings. The study of language and emotions, specifically lexical or text analysis, rests on the assumption that language features can be markers of mental health and that mental states and personal characteristics are reflected in the words people use in natural language [13]. The choice of spoken or written vocabulary conveys an individual’s thoughts irrespective of the intentions during communication [14]. Moreover, mental processes that may be out of awareness influence how an individual selects the words to be used by revealing itself through lexical leakages. Regardless of the contextual meaning of words, the selection, per se, is determined by a mental preoccupation with a particular theme. In other words, an individual, possessing a certain thought pattern (eg, I am worthless), may reveal these thoughts through spoken and written language.

In studying depression and its symptoms, everyday language reveals the cognitive mechanisms activated [15]. Depression involves negative schemas that guide attention towards and enhance recall of negative experiences [16]. Continually being reinforced, active negative schemas progress and develop into negative views of the self, the world, and the future. However, at times, latent or dormant negative schemas expose themselves only upon the experience of a stressor or cognitive load/task that interferes with a person’s attempts to reduce unwanted negative thinking [17]. Either way, schemas control one’s beliefs and automatic thoughts which are accessible and are manifested as words indicating negative emotions and negative thinking [18] or sometimes suicide and death [19,20]. According to the Self-Focus Model of Depression, depressed or depression-prone individuals tend to ruminate and interpret events in terms of themselves [21]. Depressed individuals generally engage in higher levels of self-focus resulting in a higher activation of negative self-schema. The depressive self-focusing style then maintains and exacerbates the disorder or heightens vulnerability. Modalities, such as increased social media activity, raised negative affect, high self-attentional focus, heightened relational and medicinal concerns, and greater expression of religious involvement, may characterize and signal depression onset [22].

Worldwide, mobile phone ownership has reached more than 6 billion [23]. Daily life events and digital behaviors, such as Internet usage, SMS text messaging (short message service, SMS), online chatting, blogging and social media access, are converging around mobile devices [24,25], making the technology a highly integral part of day-to-day activities, specifically in the Asia Pacific. In the Philippines, mobile phones with app capabilities are increasingly utilized for communication and entertainment [26], especially in the 18 to 24 age bracket [27].

The popularity of mobile technology is harnessed in the delivery of health care and information via mobile health (mHealth) [28]. Mobile technology’s adoption in mental health care prevents stigmatization since it attracts no undue attention to its user [29,30]. Furthermore, as it is highly integrated in daily routine, mobile devices have the capacity for Ecological Momentary Assessment (EMA), where behavior is captured in real-time or closer in time to experience, in the natural environment, and with a multitude of measurements over time [31,32]. It could also augment clinical assessment, particularly mood disturbances such as depression, and make it possible to study cognition and other processes through the analysis of daily activities, such as language use [33].

According to Rude and colleagues, language is a medium in detecting depression and depression proneness in individuals [34]. In studies involving novel avenues of communication, activities in social networking sites (SNS) could signal or reveal symptoms of depression or expose at-risk and fatal suicidal behaviors: disclosing feelings of depression through status updates on Facebook [35,36], decreasing levels of social activity accompanied by increasing levels of negative emotions, and interest in medication [22,37], reading more tips and facts about depression [38], hinting at signs of hopelessness and meaninglessness, even somatic pains through Facebook [39], posting ruminating behaviors on Twitter [40], and expressing suicidal feelings or communicating suicide-related behavior on social media [41].

**Figure 1:**
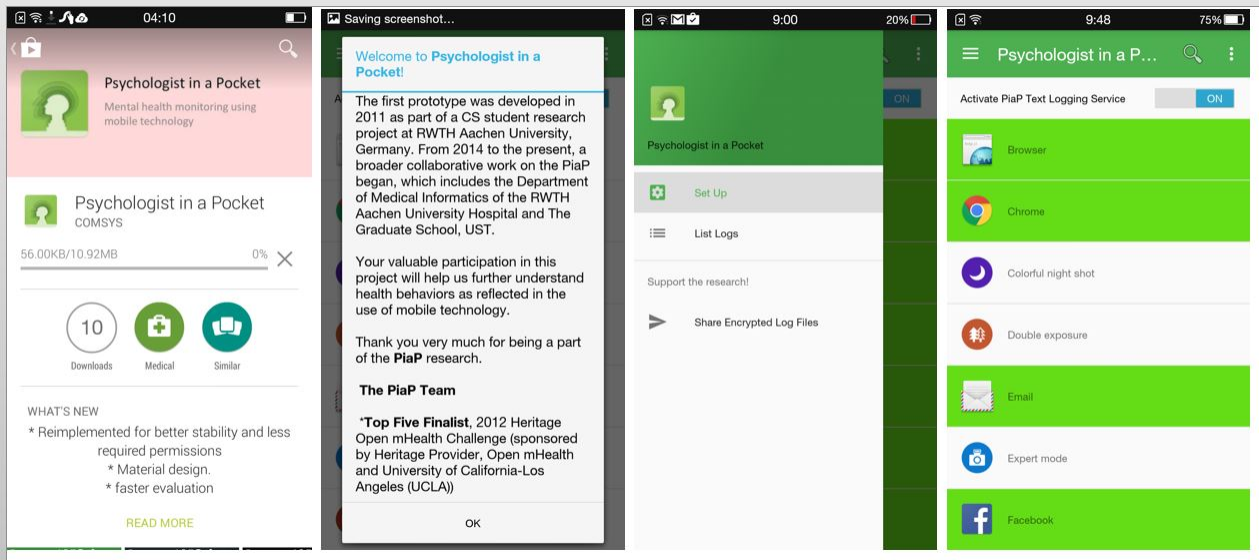
Screenshot of the version of Psychologist in a Pocket currently being tested and discussed in this paper.

Tapping into the pervasive presence of mobile technology in daily functioning and its utilization in mental health care, this paper presents the development and validation of a depression lexicon for the mobile mental health app Psychologist in a Pocket (PiaP), an open source Android-based app designed to detect symptoms of depression using EMA [42]. PiaP is envisioned to be a novel screening approach for depression symptoms to augment traditional clinical assessment (Figure 1). It is not intended to diagnose or to replace mental health professionals. Specifically, the objectives of the study are (1) to develop a depression lexicon using both bottom-up and top-down approaches for automated text analysis functions; and (2) to validate this lexicon in terms of its content or adequacy in representing its domain.

## Methods

### Lexicon Development

The PiaP app is designed to be a measurement tool for depressive symptoms employing a novel method by performing text analysis directly on a mobile device. One of the first stages in developing a test or a measurement tool is to create items to represent the domain. In this case, the initial step in PiaP’s development is to generate a lexicon of keywords to represent depressive symptoms. While the development of the lexicon is purported for the text analysis function of PiaP, the depression lexicon or database of depressive words and phrases may be used in any type of text analysis or sentiment analysis that could be computer or mobile-based.

In building a lexicon of a particular subject or domain such as depressive symptoms, the top-down and bottom-up approaches may be utilized in order to represent an entire domain [43]. The top-down approach, also called the gold-standard approach, starts with a domain and from there, fills in details that would be descriptive of that domain using expert knowledge. It establishes the principles with which the domain may be communicated, and for each principle, determines on the basis of competence or observational data, what words are employed to communicate the same or a particular meaning. The bottom-up approach starts with individuals and their linguistic elements and then records and analyzes each word while testing if the same words or expressions always have the same meaning.

In building the depression lexicon, both the gold-standard descriptions (ie, DSM and ICD classification systems) and the accounts of the individuals themselves who experience the symptoms were utilized to ensure that the lexicon encompasses most of the domain. Furthermore, the language culture of the target population must be considered to make certain that the lexicon reflects both the traditional and slang ways of symptom expression.

### Building of Categories

The depressive symptoms listed and described under Depressive Episode in the Diagnostic and Statistical Manual of Mental Disorders, Fifth Edition (DSM-5) [44] and in the ICD-10 Classification of Mental and Behavioural Disorders: Clinical Descriptions and Diagnostic Guidelines [45] were the basis for the categories of the depression lexicon (Table 1). We created keywords under each category to represent their domain or construct. In other words, DSM-5 depressed mood/irritable mood symptom and ICD-10 depressed mood symptom correspond to the PiaP category termed Mood which has keywords encompassing and representing symptoms of depressed mood or irritable mood.

**Table 1:** DSM-5 and ICD-10 codes and their category representation.

Category	DSM-5	ICD-10
Mood	Depressed mood	Clearly abnormal depressive mood
Interest	Markedly diminished interest or pleasure in all or almost all activities	Marked loss of interest or ability to enjoy activities that were previously pleasurable
Appetite and weight	Significant weight loss or gain, or increase or decrease in appetite	Changes of appetite (decrease or increase), with the corresponding weight change
Sleep	Sleep problems	Sleep alterations of any kind
Psychomotor agitation	Psychomotor agitation or retardation	Changes of psychomotor activity, with agitation or inhibition
Psychomotor retardation	Psychomotor agitation or retardation	Changes of psychomotor activity, with agitation or inhibition
Fatigue	Fatigue or loss of energy	Lack of vitality or increase of fatigability
Guilt and self-esteem	Feelings of guilt, worthlessness, negative self-appraisal	Disproportionate self-reproaches and feelings of excessive guilt or inadequacy; loss of confidence and self-esteem and feelings of inferiority
Concentration	Diminished concentration or indecisiveness	Complaints about or decrease of the ability to concentrate and think, accompanied by a lack of decision and vacillation
Suicide	Recurrent thoughts of death or suicidal ideation	Recurrent thoughts of death or suicide or any suicidal behavior
Anxiety	Anxiety	Anxiety
Alcohol and substance use	Substance abuse	Excessive consumption of alcohol
Histrionic behavior	Histrionic behavior	Histrionic behavior

Symptoms, such as anxiety, substance use, and histrionic behavior, which are associated symptoms or features of depression according to these diagnostic systems, were also included in the lexicon. The keywords in each category were gathered using the approaches of focus group discussions with college students, interviews with mental health professionals, review of depression scales, and spelling variation of keywords generated.

### Focus Group Discussions

Focus group discussions were conducted with 76 college students with a mean (SD) age of 17.28 (1.14) and 61% female (46/76) whose depression symptoms based on Beck’s Depression Index II (BDI-II) scores ranged from 14 (mild) to 63 (severe) (Table 2). The purpose of the focus group discussions was to gather words, expressions, and symbolic representations of depression. This bottom-up process of lexicon building creates an inclusive representation of the linguistic ways people use to express the experience of depression. Participants were randomly assigned to 1 of the 7 focus group discussion sessions (10 to 11 participants per session), each lasting for 45 to 60 minutes. Before the conduct and recording of each session, 2 of the researchers acting as moderator and note-taker, provided a quick briefing of the study. Informed consent from participants was also obtained prior to the sessions.

**Table 2:** Focus group discussion participants (N=76).

Variables	Categories	n (%)
Gender			
	Female	46 (61)
	Male	30 (39)
Age, years		
	16	18 (24)
	17	29 (38)
	18	17 (22)
	19	5 (7)
	20	2 (3)
	22	1 (1)
	Not specified	4 (5)
BDI-II score		
	Mild	32 (42)
	Moderate	29 (38)
	Severe	15 (20)

Questions centered on how the participants define and describe their depression, what words they typically use to express depression in mobile text inputs and in social media, and how they recognize the signs of depression in another person’s text input or social media activity. At the end of each discussion, participants were presented with a set of emoticons and emojis depicting negative emotions. They were asked to choose the emoticons and emojis they frequently used to describe the emotions and feelings associated with the experience of depressive symptoms.

All responses were transcribed verbatim into Excel and examined by 3 of the researchers for analysis and synthesis of possible themes. Transcripts were coded according to identified patterns and relationships.

### Interviews With Mental Health Professionals

As part of the top-down or gold standard method of lexicon building, 5 selected mental health professionals, 2 psychiatrists, 2 clinical psychologists, and 1 counselor facilitator of a support group for depression and substance use, were interviewed. The purpose of the interview was to determine how depressive symptoms among Filipino adolescents are linguistically expressed via mobile technology. Specifically, questions revolved around typical words and phrases of a patient or client who experiences depressive symptoms, ways to identify depressed individuals through novel communication like social media, and feasibility of mobile apps for depression.

The selection criteria of the mental health professionals for interview included (1) more than 10 years experience in their chosen fields (mean 19 years); (2) are officially recognized by the Philippine Regulatory Commission as a licensed psychologist, psychiatrist, or counselor; and (3) have been working with adolescents and young adults suffering from depression. Letters of request for interview were sent to 12 mental health professionals. Of those, 5 agreed to participate in the interview. They were also requested to confirm the findings and the generated keywords from the focus group discussions. Each interview was tape recorded and transcribed verbatim into Excel to infer the main points.

### Review of Depression Scales

Another top-down approach in lexicon building was to inspect established and psychometrically sound measurement tools for depression. Words that depict depressive symptoms and variables related to depression, such as negative affect, were extracted from 18 scales, questionnaires, and inventories (Textbox 1). Nouns, verbs, adjectives, and adverbs that relate to depressive symptoms were gathered and frequencies were computed for each keyword found in these sources.

**Reviewed scales for depression and related constructs.**

Reviewed scales

1. Beck Depression Inventory II (BDI-II) (Beck, Steer, and Brown, 1996)

2. Center for Epidemiologic Studies-Depression (CES-D) scale (Radloff, 1977)

3. Affect Balance Scale (ABS) (Bradburn, 1969)

4. Experiences of Low Mood and Depression (ELMD) questionnaire (Peyton and Critchley, 2005)

5. Hospital Anxiety and Depression Scale (HADS) (Zigmond and Snaith, 1983)

6. Patient Health Questionnaire 9 (PHQ-9) (Kroenke and Spitzer, 2002)

7. Major Depression Inventory (MDI) (Bech, 1997; Bech and Wermuth, 1998)

8. Inventory to Diagnose Depression (IDD) (Zimmerman and Coryell, 1987)

9. Depression Anxiety Stress Scales (DASS) (Lovibond and Lovibond, 1995)

10. Crandell Cognitions Inventory (CCI) (Crandell and Chambless, 1986)

11. Dysfunctional Attitudes Scale (DAS) (Weissman and Beck, 1978)

12. Situational Self Statement and Affective State Inventory (SSSASI) (Harrell, Chambless, and Calhoun, 1981)

13. Smith Irrational Beliefs Inventory (SIBI) (Smith, 2002)

14. Automatic Thoughts Questionnaire (ATQ) (Hollon and Kendall, 1980)

15. Cognitive Triad Inventory (CTI) (Beckham, Leber, Watkins, Boyer, and Cook, 1986)

16. Cognition Checklist (CCL) (Beck, Brown, Steer, Eidelson, and Riskind, 1987)

17. Irrational Beliefs Inventory (IBI) (Koopmans, Sanderman, Timmerman, and Emmelkamp, 1994)

18. Young’s Early Schemas Questionnaire (YSQ) (Young, 2003)

### Spelling Variation of Keywords

Young Filipinos typically abbreviate or change the spelling of certain words and mix Filipino/Tagalog and English words whilst communicating. This even coined the terms “textolog” and “tag-lish”. Written language in communication modes such as text messages or SNS has unique characteristics, thus a detection tool must encompass the peculiarities of language expression of different cultures.

To take into consideration the unique culture of Filipinos in inputting text in mobile devices, 500 printed forms containing the list of all main keywords and their derivatives (eg, verb changes) gathered from the previously mentioned methods were distributed randomly to students in 4 universities and colleges (3 in Metro Manila and 1 in Central Luzon). Instructions were to provide at least 3 abbreviations or spelling variations of the keywords. Through convenient selection, data was only considered from 328 students who were able to complete the forms within 2 weeks.

### Categorization of Keywords

In the top-down approach, all keywords are generated based on the previously defined categories and are therefore inherently categorized. For the bottom-up approach, all keywords generated by the focus group participants need to be classified into one of the existing categories. This task was initially performed by 2 of the authors (PGFC and RMR). Keywords were distributed and grouped according to the symptom they represent. This keyword categorization was then subjected to content validation by 8 experts.

### Content Validation by Experts

According to Kaplan and Saccuzzo [46], the adequacy of representation of the construct or domain the test or tool is designed to measure is reflected by its content-related evidence for validity. Content validation is one the psychometric procedures that index a test’s validity or its ability to measure what it purports to measure. A typical method of content validation involves multiple judges rating each item in the test in terms of its relevance to the content [47].

Content validation was conducted to ensure that each keyword belonged to the correct category. Mental health practitioners (N=22) comprised of licensed clinical psychologists, psychiatrists, guidance counselors, and psychology instructors were requested to validate the list. Of those, 36% (8/22) agreed to conduct the content validation. These experts (1) have at least 10 years experience in their chosen fields (mean 17.13 years); (2) are officially recognized by the Philippine Regulatory Commission as licensed psychologists, psychiatrists, or counselors; (3) work with adolescents and young adults with mental health problems; and (4) are currently professionally involved in at least 2 fields such as in the academe and clinical practice.

Using Lawshe’s content validity ratio (CVR) in deriving the content validity index (CVI), the experts rated whether the keywords were “essential”, “useful but not essential”, or “not necessary” to the performance of the construct [48,49]. CVR per keyword is depicted by the ratio of the number of experts indicating a keyword as “essential” over the total number of experts. The greater the number of the experts indicating a keyword as “essential”, the greater is the keyword’s content validity. For the 8 validators, each item or keyword must reach a CVR equal to or more than 0.75 to be included in the lexicon [49,50]. The CVI is the mean CVR of all the retained keywords representing the commonality of judgments regarding the validity of the lexicon. The overall content validity is considered high since the value of the CVI approached 1. For the CVRs that did not reach the acceptable CVR level, further selection of keywords to be retained is still possible. For every keyword, 2 judgement points were awarded per acceptance by a validator or when a validator rates the item as “essential”. Afterwards, mean judgement points per keyword were calculated. If a keyword’s CVR is between 0.0 and 0.5 and the mean of judgment points is greater than 1.5, then the keyword is accepted [51].

## Results

### Lexicon Development

#### Building of Categories

Since the depression lexicon categories were based on the symptoms described in the DSM-5 and ICD-10, the current lexicon is comprised of 13 symptom categories: mood, interest, appetite and weight, sleep, psychomotor agitation, psychomotor retardation, fatigue, guilt and self-esteem, concentration, suicide, anxiety, alcohol and substance use, and histrionic behavior.

#### Focus Group Discussions

From the focus group discussions, words typically used to express depressive symptoms were gathered. As seen in Table 3, 27% (21/78) of the responses indicate being “sad” and “lonely” as major descriptions of people with depression. Specifically, the words “sad”, “unhappy”, and “loneliness” as well as the word “loner” were used most often. In Filipino/Tagalog, some describe the experience of depression as having no focus (“tulala”, “lutang”, “malayo ang iniisip”) and as being disturbed or messed up (“wala sa sarili”).

**Table 3:** Descriptions of depression (N=78).

Responses	n (%)	Cumulative %
Sad; lonely; unhappy	21 (27)	27
No focus; disturbed	11 (14)	41
Isolation; lack of interest; low interaction	7 (9)	50
Sleep problems	7 (9)	59
Hopelessness; loss of meaning in life	6 (8)	67
Fatigue; stressed	5 (6)	73
Pessimism	4 (5)	78
Uneasiness; instability	4 (5)	83
Moody	3 (4)	87
Emotional	2 (3)	90
Eating problems	2 (3)	92
Low self-esteem	2 (3)	95
Suicidal	1 (1)	96
Anxiety	1 (1)	97
Have no emotional support	1 (1)	99
Pretending to be happy	1 (1)	100

As seen in Table 4, 56% (37/66) of the responses suggest that people who are depressed reveal their depression online. SNS serve as emotional outlets, thus allowing them to verbalize their feelings and release their sadness, frustrations, and problems. This capability to express emotions is somewhat foreshadowing, especially for those about to become clinically depressed. However, 20% (13/66) of the responses indicate that depression may be disguised by expressing opposite emotions. Still, 9% (6/66) of the responses suppose that people with depression use SNS and text messages as a signal and a means to reach out for help.

**Table 4:** Revealing own depression in texts and social media (N=66).

Responses	n (%)	Cumulative %
Social network sites are emotional outlets	37 (56)	56
Hide depression or show the opposite of depression	13 (20)	76
Social network sites and text messages signal help	6 (9)	85
Sadness and troubles are expressed via social network sites (mildly depressed > severely depressed)	6 (9)	94
Seek attention via social network sites or text messages	3 (5)	99
Simply follow trends	1 (1)	100

As seen in Table 5, 35% (18/50) of the responses indicate that they are able to recognize people with depression from SNS posts and text messages through the use of sad words. These may include not only personal messages but also quotations or lyrics of sad songs. In addition, 24% (12/50) of the responses claim that they do notice someone experiencing depression, particularly via the changes in behavior and topics of messages as observed in the history of postings and group messages or messages sent to predefined contacts, usually consisting of close friends. However, depressed people, even though they may post texts online, are thought to be difficult to talk with and are hesitant to open up about their depression in face-to-face interactions, as 8/50 (16%) responses point out that people with depression may be very aloof and keep quiet about their condition. They may not answer truthfully when asked directly about their feelings (despite having posted sad texts online). Depressed people have difficulty expressing themselves when confronted but would post messages, seemingly asking people for help or putting forth a message and reaching out for people.

**Table 5:** Recognizing others’ depression in texts and social media (N=50).

Responses	n (%)	Cumulative %
Presence of “sad” words	18 (36)	36
Change in behavior or topics	12 (24)	60
Asking for help	8 (16)	76
Only recognized if the reader is in a close relationship with the depressed individual	5 (10)	86
Postings or text messages as insincere or inconsistent	4 (8)	94
Might be misinterpreted	2 (4)	98
Only mental health professionals can recognize	1 (2)	100

#### Interviews with Mental Health Professionals

From the interviews with 5 mental health professionals, it was confirmed that language in any form, written or verbal, is able to reveal signs of depression. The following points have emerged. First, clients or patients usually tell them when they are depressed. Some of the words, phrases, or sentences typically used by the clinically depressed are “can’t eat”, “can’t sleep”, “just going through the motions”, “no purpose or meaning”, “not interested”, “I’m tired all the time”, “I feel worthless”, “guilty”, “It’s not going to work whatever I do”, Nothing will change“, ”My head/stomach/muscles ache/s“, I’m losing weight”, and “I’ll never get well”.

Second, description of symptoms, such as that of the DSM and depression tests, may differ from the exact expressions of the individual when communicating with others. For instance, suicidal thoughts among the participants may be expressed as “Lord, please take my life”. Another example is that some symptoms described in the diagnostic systems such as psychomotor retardation may be difficult to translate into words but can be represented by specific actions in sentences such as “I find it tiring to dress up” or “I have a hard time making decisions”. This suggests the possibility that the symptoms, as indicated in items of traditional paper-and-pencil tests, may be general descriptions or collective terms for the experiences of the individual. Personal descriptions of depression may differ from the words used in these tests.

Third, current and contemporary language styles play an important role in developing a lexicon. The use of emoticons and emojis in expressing feelings is a trend in SNS and text messaging. Depressed mood, for example, may be expressed using “:-(”, which may mean “sad” or “lonely”.

Finally, it is feasible to express depression through text messages, blogs, or SNS. A clinical psychologist with 13 years of experience handling young adults mentioned that such apps are new technologies adolescents use; however, to detect depression through these apps, developers must consider all the possible words that maybe be used. Due to the integration of mobile devices in daily activities, a mobile app for depression may be useful for individuals who need help.

#### Review of Depression Scales

Keywords gathered from the review of depression scales were mostly statements in the first-person point of view. The words were grouped according the depressive symptoms, as stated in the DSM-5 and ICD-10 (Table 6). Words with the highest frequency convey either guilt or lowered self-esteem. This is followed by depressed mood and decreased interest or pleasure. However, no items related to alcohol consumption or substance use and abuse were found in these scales.

**Table 6:** Keyword frequency from depression scales (N=449).

Depressive symptoms	Frequency (%)	Cumulative, %
Feelings of guilt and low self-esteem	148 (33.0%)	32.96
Depressed mood	103 (22.9)	55.90
Loss of interest or pleasure	40 (8.9)	64.81
Anxiety	35 (7.8)	72.61
Psychomotor agitation	27 (6.0)	78.62
Fatigue	23 (5.1)	83.74
Increase or decrease in appetite or weight	20 (4.5)	88.20
Diminished concentration	17 (3.8)	91.98
Sleep problems	15 (3.3)	95.32
Suicidal thoughts and behavior	12 (2.7)	98.00
Psychomotor retardation	8 (1.8)	99.78
Histrionic behavior	1 (0.2)	99.78
Consumption of alcohol	0 (0.0%)	100.00

#### Spelling Variation of Keywords

A total of 823,869 spelling variations of keywords were generated. The spelling variations were extended by the researchers by determining all possible combinations of individual words within a keyword (Table 7).

**Table 7:** Sample spelling variations of keywords.

Category	Keyword	Variation 1	Variation 2
Depressed Mood	Can not stop crying	Cant stp crying	Cnt stop cryin
Interest	Want to detach	Wnt 2 detach	Wanna detach
Sleep	Trouble falling asleep	Trouble fallng asleep	Trble fallin aslp
Suicide	Take my life	Tke my life	Take my lyf
Guilt/self-esteem	I am worthless	I’m wrthless	Im worthlss

#### Categorization of Keywords

The PiaP lexicon development phase yielded a database consisting of 13 categories patterned after DSM-5 and ICD-10 symptom criteria for depressive episodes. The categorization and frequency of keywords per category is shown in Table 8. For the lexicon draft, we were able to gather 1762 main keywords, 9655 derivatives with different tenses or arrangement of words of the main keywords, and 823,869 spelling variations. The keywords included negatively-valenced words like “sad”, “unworthy”, or “tired” which are almost always accompanied by personal pronouns such as “I”, “I’m” or “my” and in Filipino “ako” or “ko”.

**Table 8:** Categorization of keywords.

Categories	Main Keywords, n	Derivatives, n	Spelling variations, n	Total, n
Mood	241	1582	63,340	65,163
Interest	129	1035	107,417	108581
Appetite and weight	216	1357	19,032	20,605
Sleep	162	786	187,365	18,8313
Psychomotor agitation	174	750	12,957	13,881
Psychomotor retardation	74	431	16,160	16,665
Fatigue	112	424	14,964	15,500
Guilt and self esteem	180	1000	30,067	31,247
Concentration	165	753	239,291	240,209
Suicide	90	635	58,732	59,457
Alcohol and substance use	63	315	20,875	21,253
Anxiety	112	399	45,251	45,762
Histrionic behavior	44	188	8418	8650
Total	1762	9655	823,869	835,286

### Content Validation by Experts

The CVR per keyword was computed and averaged to the CVR per PiaP category. The categories of appetite and weight (CVR=0.98), suicide (CVR=0.97), and guilt and self-esteem (CVR=0.94) obtained the highest ratios (Table 9). On the other hand, the lowest ratios were for the psychomotor retardation (CVR=0.80) and psychomotor agitation (CVR=0.78) categories. The mean of all CVRs yielded an overall CVI of 0.90. For the 8 validators, an acceptable CVR per item or keyword should be greater than or equal to 0.75. The overall CVI was determined to be 0.90.

The content validity testing resulted to 1498 keywords, 8911 derivatives of main keywords, and 783,140 spelling variations, with a total of 793, 553 keywords which comprise the test-run version of the PiaP lexicon (Table 10).

**Table 9:** CVR per keyword category.

PiaP category	CVR
Mood	0.86
Interest	0.93
Appetite and weight	0.98
Sleep	0.92
Psychomotor agitation	0.78
Psychomotor retardation	0.80
Fatigue	0.89
Guilt and self-esteem	0.94
Concentration	0.93
Suicide	0.97
Alcohol and substance use	0.91
Anxiety	0.89
Histrionic behavior	0.92
CVI	0.90

**Table 10:** Categorization of keywords after content validation.

Categories	Main keywords, n	Derivatives, n	Spelling variations, n	Total, n
Mood	181	1375	52,130	53,686
Interest	113	1011	102,081	103,205
Appetite and weight	213	1353	19,018	20,584
Sleep	148	743	183,123	184,014
Psychomotor agitation	129	544	8993	9666
Psychomotor retardation	51	366	12,578	12,995
Fatigue	103	420	15,080	15,603
Guilt and self-esteem	173	984	29,837	30,994
Concentration	135	668	229,375	230,178
Suicide	86	612	58,355	59,053
Alcohol and substance use	53	315	20,760	21,128
Anxiety	82	359	44,065	44,506
Histrionic behavior	31	165	7745	7941
Total	1498	8915	783,140	793,553

## Discussion

### Principal Findings

The current study involves lexicon development and content validation for a lexicon of depressed words to be used in mobile mental health apps, such as the PiaP. In lexicon development, we found that (1) language used in contemporary avenues such as social media and mobile technology serve as channels for expressing depression-associated emotions while subtly asking for help and avoiding stigmatization; (2) depressive language consists of words with negative undertones plus pronouns pertaining to the self; and (3) gold standard depiction of depressive symptoms, such as that of tests and diagnostic systems, may be different from the exact verbal expressions of the individual experiencing it. In addition, the uniqueness of a group or of a specific culture’s language expressions should be taken into account. A measurement tool, in this case a depression lexicon, with a high content validity shows its potential in detecting the domain, which are depressive symptoms.

The focus group discussions with the college students pointed to the idea that with the use of social media incorporated in their mobile phones, it becomes easier to simultaneously express one’s self and to relate and reach out to others. Language use is analyzed in detecting depression and depression proneness as shown in a study using an essay writing activity with students expressing their thoughts and feelings about college [34]. With social media offering a platform where language can be studied, it is even possible to identify people with depression in their free-texts and novel ways of communication [22,37]. College students’ disclosure of feelings or emotions related to depression can likewise be determined through status updates and postings on Facebook [35]. Students who may be experiencing depression spend more time on Facebook and could reveal more about themselves online rather than interpersonal communication [36]. Previous studies also support our focus group discussion findings in terms of recognizing depressive symptoms through increasing levels of negative emotions [22], reading more tips and facts about depression [38], hinting at signs of hopelessness and meaninglessness and posting ruminating behavior, and expressing suicidal feelings or communicating suicide-related behavior on social media [39-41].

Depressive language consists of words with negative undertones plus pronouns pertaining to the self as evidenced by keywords gathered from focus group discussions, interviews with professionals, and depression tests. Our interviews and discussions with mental health professionals confirmed that depressed clients often have a negative description of themselves, of their situation and environment, and of their lives and futures that may be revealed in social media. These findings are supported by Beck’s cognitive theory [16], and Pyszczynski and Greenberg’s self-focus model [21], which describe individuals experiencing depression as using more words with negative connotations and often making self-references when sharing their experiences. Whenever people are posting in social media, sending emails or text and instant messages, reacting to a friend’s status message, or using search browsers, the schema, negative or positive, with which their minds operate can be observed regardless of the purpose or intention of communication through the words they choose to type in their mobile devices.

Gold standard depictions of depressive symptoms, such as that of tests and diagnostic systems, may be different from the exact verbal expressions of the individual experiencing depression. This is supported by a study by Neuman and colleagues when they created the Pedesis system by gathering depressive words as experienced and expressed by individuals who have depression (eg, blogs and social media) [43]. People have vast ways of expressing depression, which may not be encompassed by a list created by experts or by diagnostic systems, such that the classification provided by the DSM or ICD. Even descriptions made by mental health professionals mainly consist of symptoms and signs rather than expressions of depression [43]. Culture or generational differences makes it even more complicated when trying to make a representation of expressing depression in words. With novel platforms for communication such as mobile technology and social media, it is indeed challenging to encompass the variety of ways people may express themselves [52,53]. A guideline of what to look for is provided by the gold standards but it is in the examination of the lexical expressions of people experiencing depressive symptoms that depression can be adequately characterized [43,52,53]. In addition, the current study’s findings show the texting behaviors of Filipinos, particularly among college students, involve abbreviating or changing the spelling of words, which, to an outsider appears almost incomprehensible. The inclusion of abbreviations or spelling variations increased the bulk of keywords, but since text analysis relies on the lexicon for detecting the symptoms, disregarding this cultural and generational peculiarity, would make it impossible to capture relevant depressive language.

The content validation procedure is a measure of validity of the lexicon in detecting words that can be related to depressive symptoms using text inputs. The data suggests that depressive language can express the majority of the symptom categories. This might indicate that symptoms more cognitive in nature are better expressed in verbal behavior. Schemas supporting such symptoms influence automatic thoughts that become observable in language use or choice of words. However, the identification of motor or physiological symptoms, such as psychomotor retardation and agitation, was less obvious. These symptoms are likely to be better observed in bodily movements and gestures and reported by others. Individuals who exhibit these symptoms may not be able to notice them themselves.

With its sound content validity, the depression lexicon adequately represents words that can be related to depressive symptoms and may be able to help in the detection of symptoms using text analysis in the mobile mental health app PiaP. An ongoing study involves a test trial of PiaP with selected college students and will investigate the individual keywords and their ability to discriminate and detect individuals who may have depressive symptoms through the psychometric process of item analysis. In addition, data will be used to calculate the internal consistency of the depression lexicon. Further trials with college students will also determine the normative structure of the population chosen.

### Significance

The study has significance in the fields of mental health (eg, clinical psychology, psychiatry, counseling) and health informatics by seeking to deliver not just a psychometrically sound instrument but also a highly usable tool for the assessment of depression. Mobile phones with app capabilities have enabled ordinary individuals to do almost anything and everything with ease. The flexibility and proliferation of mobile phone software programs have allowed professionals in many fields in science and medicine to accomplish tasks related to gathering pertinent physiological data from their patients to aid diagnosis and help monitor the progress of physical or pharmacological treatment [54]. Psychologists, psychotherapists, guidance counselors, and other mental health professionals may take advantage of this timely approach to monitor their clients, patients, or students.

The field of mHealth has much potential in developing countries [29,25], even more so mobile mental health, which in some places is still in its infancy. The mental health profession has been heavily relying on traditional and conventional assessment tools such as pen-and-paper tests, observations, and interviews to gather data. Mobile mental health solutions such as the PiaP may contribute to the diagnosis process in a proactive way as well as monitor current symptomatology. Together with other technology-based tools that allow EMA, our approach may help provide increased statistical power of datasets gathered due to the amount of observations available. With the introduction of the new technique in screening for depressive symptoms described in this paper, the mental health field may begin exploring other possibilities like providing treatment aids with the use of mHealth technology. This may be accomplished in collaboration with experts and professionals in mobile technology and computer science.

Lastly, this study is intended to support suicide prevention among students by contributing a dependable and suitable tool for guidance counselors and mental health experts in the academe. Although depressive signs and symptoms may or may not lead to suicide, it is advantageous for the early detection of signals (such as non-fatal self-harm behaviors) to facilitate prompt action.

### Prior Works in Lexicon Development

The lexicon development used in the PiaP has taken into consideration the processes employed in creating earlier lexica from other researches to be able to provide a comprehensive representation of the depressive language used by college students.

First, the current depression lexicon underwent an exhaustive process using both the top-down and bottom up processes. Pedesis [43] was built using the bottom-up process only, by harvesting the Web for metaphors in which the target terms (ie, depression) embedded are used by individuals who may or may not be experiencing depressive symptoms. De Choudhury et al [22] built a lexicon of depression-related terms based on the social media platform Twitter. Emotex [52] includes unigrams, emoticons, negations, punctuations and hashtags, emotions and moods in social media language, and utilized words from regular individuals to build their lexicon. On the other hand, in the development of the current lexicon used by PiaP, descriptions from mental health professionals and established literature in the field of emotion studies as well as words depicting depression in diagnostic system such as DSM and ICD were also included.

Second, the developed lexicon is culturally sensitive, having included language and jargon used by a specific group of people, particularly college students. In addition, the app also analyzes emoticons in SNS and abbreviations or spelling variations of text inputs which are novel ways of expressing feelings and emotions. De Choudhury et al [22] utilized a dataset which classified emotions in various technological contexts and used a list of mood words from a blogging website. It also incorporated various lexica for positive and negative emotions and for basic emotions, but novel words used in social media communication such as emoticons, emojis, or abbreviations were not included. In Xue and colleagues’ study [53], although the tweets they examined were a combination of English and Chinese words, the lexicon they built only included English words pertaining to stress, negative emotion, degree, and negation. However, Chinese tweets needed to be translated to English before submitting to text analyses.

Third, the initial validation of the lexicon offers content-related evidence for its psychometric property of validity. Pedesis [43] employs inter-rater or inter-judge reliability testing; however, they did not test for domain representativeness of the words in the lexicon itself.

### Current and Future Work

Currently, PiaP research in the Philippines is working with college students to establish construct validity against tests and scales for depression and related constructs such as BDI-II, CES-D scale, Satisfaction with Life Scale, and Affect Balance Scale. This approach may show the possible correlations with constructs similar or related to depressive symptoms. Also, PiaP is being administered to selected college students to gather data for item analysis which will examine the usage of words and its ability to discriminate between individuals who are depressed or non-depressed. Furthermore, PiaP data will be used to establish normative structure for college students. In Germany, a third depression lexicon (in German) is being initialized. In addition, work is being performed on the behavioral indicators of depression using sensors and voice markers. Future research will also include the incorporation of physiological test plug-ins such as mobile electroencephalography (mEEG).

### Conclusions

By utilizing the ubiquity and functionalities of mobile technology, the immediate detection of symptoms of one of the most prevailing clinical disorders worldwide may be improved using text analysis. The generation of this depression lexicon is exhaustive, utilizing both top-down and bottom-up approaches. The breadth of keywords used in text analysis also incorporates the characteristic expressions of depression and its related constructs by a particular culture and age group. A content-validated mobile health app, such as PiaP, may help augment early detection of depression signs by providing a lexicon, which effectively characterizes words related to depressive symptoms.

## Appendix

Multimedia Appendix 1 Paper Presentation at the 11th Biennial Conference, Asian Association of Social Psychology (AASP) and 52nd Convention, Psychological Association of the Philippines (PAP) 2015, Cebu City, Philippines, 19 -22 August.

Multimedia Appendix 2 Paper presentation at the InPACT International Psychological Applications Conference and Trends 2016, Lisbon, Portugal, 30 April - 02 May.

## Abbreviations

BDI-II: Beck’s Depression Index II
CES-D: Center for Epidemiologic Studies-Depression
CVI: content validity index
CVR: content validity ratio
DSM-5: Diagnostic and Statistical Manual of Mental Disorders, Fifth Edition
EMA: ecological momentary assessment
ESM: experience sampling method
ICD-10: The ICD-10 Classification of Mental and Behavioural Disorders: Clinical Descriptions and Diagnostic Guidelines
mHealth: mobile health
PiaP: Psychologist in a Pocket
SNS: social networking sites
