# Supplementary material for: Psychologist in a Pocket: Lexicon Development and Content Validation of a Mobile-Based App for Depression Screening
Source: JMIR Mhealth Uhealth. 2016 Jul 20;4(3):e88. doi: 10.2196/mhealth.5284 (PMC4972990; doi:10.2196/mhealth.5284)
Supplement: Multimedia Appendix 1 [file mhealth_v4i3e88_app1.pdf]

MHEALTH FOR MENTAL HEALTH

Refinement and validation of *Psychologist in a Pocket*

11th Biennial Conference, Asian Association of Social Psychology (AASP)
52nd Convention, Psychological Association of the Philippines (PAP)

21 August 2015
Waterfront Hotel, Cebu City
Philippines

***PAULA GLENDA FERRER-CHENG [1], ROANN MUNOZ RAMOS [1,2], PORTIA LYNN QUETULIO-SEE [1], TIM IX [3], JÓ ÁGILA LINK [3] & KLAUS WEHRLE[3]**

[1] The Graduate School, University of Santo Tomas, Manila, Philippines
[2] Department of Medical Informatics, RWTH University Hospital, Aachen, Germany
[3] Communication and Distributed Systems, RWTH Aachen University, Aachen, Germany

Depression

- Most prevalent clinical disorder among the youth (Whiteford, et al., 2013)
- Main cause of illness and disability in the 16-25 age group (WHO, 2014)
- In The Philippines
  - Highest incidence of depression in South East Asia 4.5 Million cases(Natasha Goulbourn Foundation, 2004)
  - Growing number of self-harm and suicide associated with mood disorders (Redaniel, Lebanan-Dalida, & Gunnell, 2011)

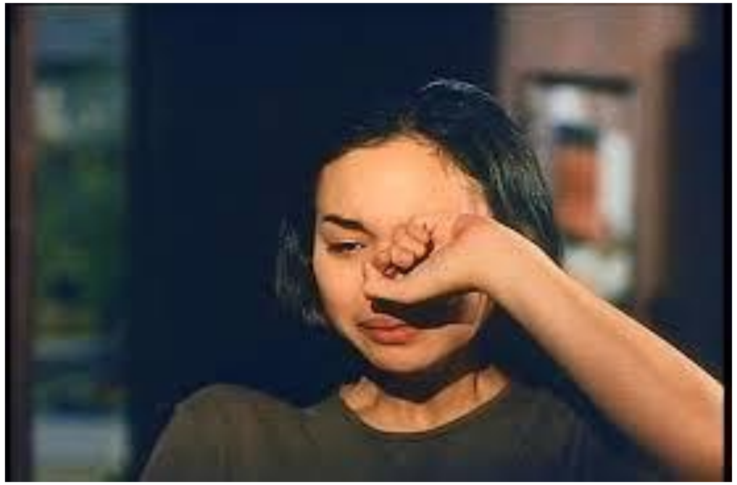

Depression among College Students

- College students:
  - poor academic performance, relationship problems, & increased drinking and smoking behaviors(Lee, Estanislao, & Rodriguez, 2013)
  - loss of weight or increase in appetite and poor sleeping patterns (Cairns et al, 2014)
  - thoughts, plans, suicide attempts and self-harm (Tiusku, 2013)
  - even in social media activities, heightened levels of negative emotions (de Choudhury et al., 2008)

Recognition: Factors to consider

- CULTURE:
  - Stigma and Losing “face” (Alonso, et al., 2008)
  - Treating depression as “normal sadness” (PDRHealth, 2014)
  - Misconceptions and Public embarrassment (Li, et al., 2014; Gulle, 2012)

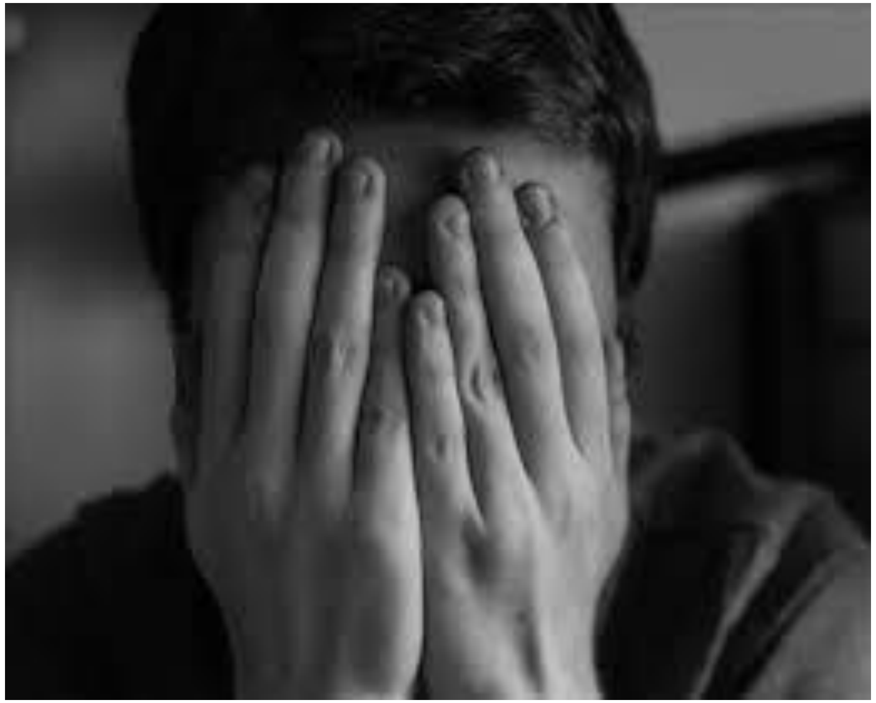

Recognition: Factors to consider

- METHOD OF DETECTION:

- Mood is transient & fluctuating

- Face-to-Face Interviews: Not the full range of events and feelings (Moskowitz & Young, 2006)

- Questionnaires & Rating Scales: Negative recall bias (Rot, Hogeneslt, & Schoevers, 2012) and Symptom- and stress-overestimation (Wenze & Miller, 2010)

Recognition: Factors to consider

- MODE OF COMMUNICATION
  - Social Media – Social Networking Sites
  - Hashtags
  - Emoticons
  - Abbreviated text inputs (“*Textolog*”)

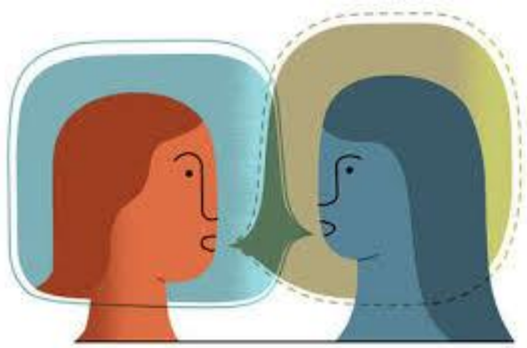

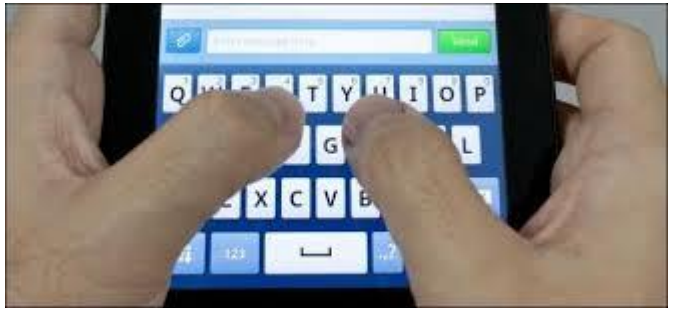

Ubiquity of Mobile Technology

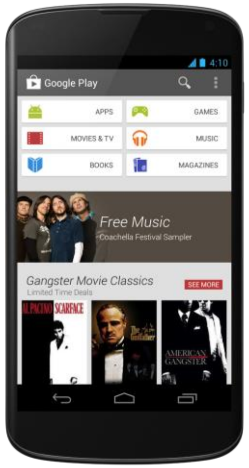

6 billion mobile users
(Gagglioli, 2013)

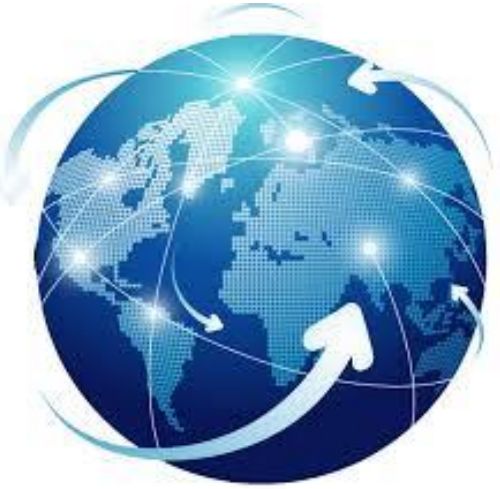

½ from Asia-Pacific
(Kemp, 2014)

- Smartphone ownership reached from 38% to 64% (Universal McCann, 2014, Wave 7)
- 83% - 18-29 y/o access Internet and social networking sites using mobile devices (Hasan, 2014; Duggan & Brenner, 2013; Labrague, 2013)

Ubiquity of Mobile Technology

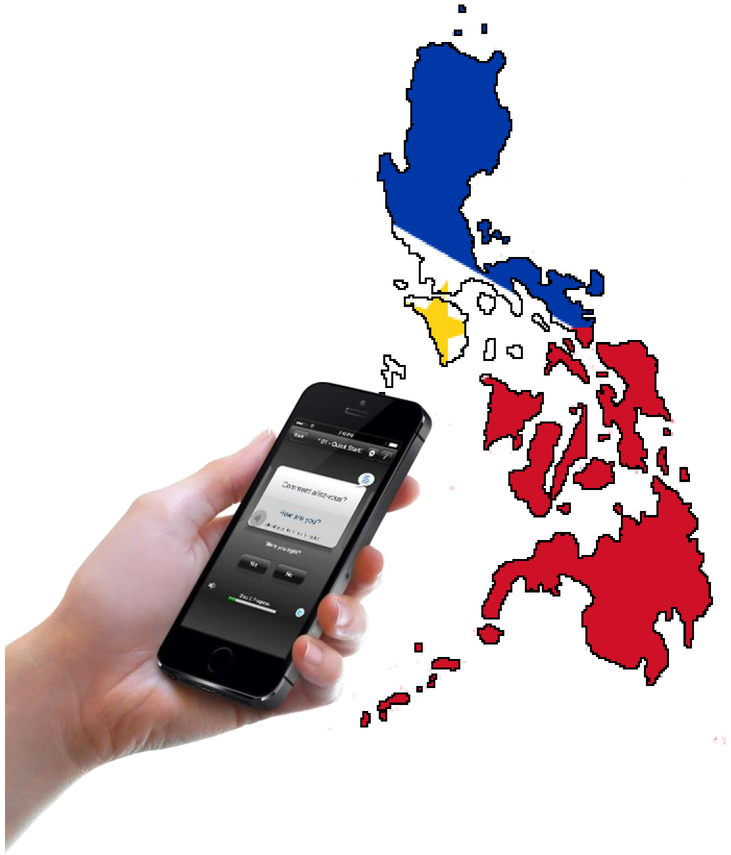

- Average time on Smartphone = 171 minutes (Nielsen, 2013)
- Use Mobile phones for internet access (Nielsen Pinoy Netizen, 2014)
- Largest age group of SNS users = 18-24 years old (Labrague, 2013)

Ubiquity of Mobile Technology

- Low cost (Doshi & Narwold, 2014)
- Highly integrated in routine (Epstein & Bequet, 2013)
- Less stigma (Brian & Ben-zeev, 2014)
- Diverse applications and functions

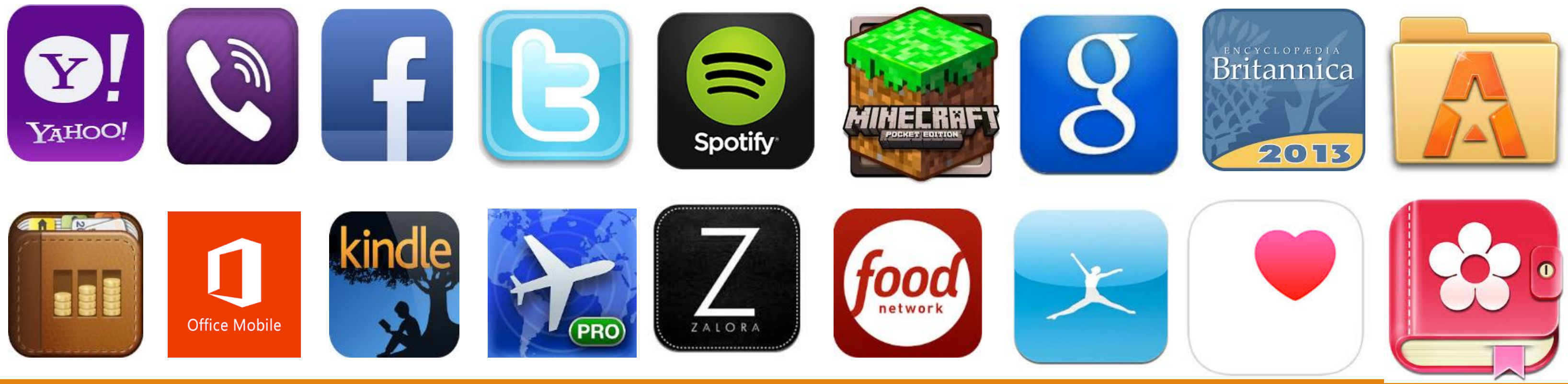

Mobile Health (mHealth)

- Delivery of health care and information through mobile technology (Varshney, 2014; WHO, 2011)

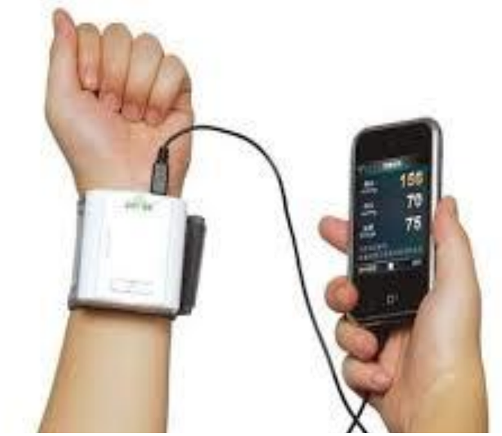

- In mental-health:
  - Monitor, treat, promote recovery
  - Examples: BeWell (sleep), StudyLife (stress), MoodRhythm (detects signs of bipolar disorder)

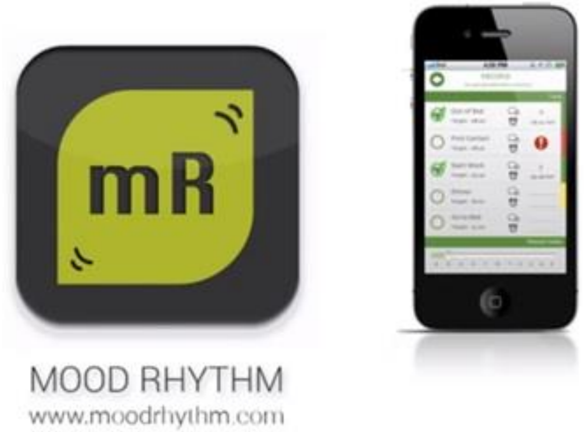

Mobile Technology in Assessment

- **Ecological Momentary Assessments (EMA)**
  - Captures behavior **Real-time, Natural environment, Multitude of assessments over time**(Stone, Shiffman, &Hufford, 2008)

- Studies cognition via analysis of daily activities like **Language**
(Neuman, Cohen, Assaf, & Kedma, 2012, Rude, et al., 2004)

Psychologist in a Pocket (PiaP)

- Ramos, Winter, Smith & Link (2012)
- **Text analysis** via **<u>Depression-lexicon</u>**
- Screens for depressive symptoms
- Privacy and Security: Local evaluation
- Reporting Function: Prompt 3rd Party which is user-defined (eg. psychologist)

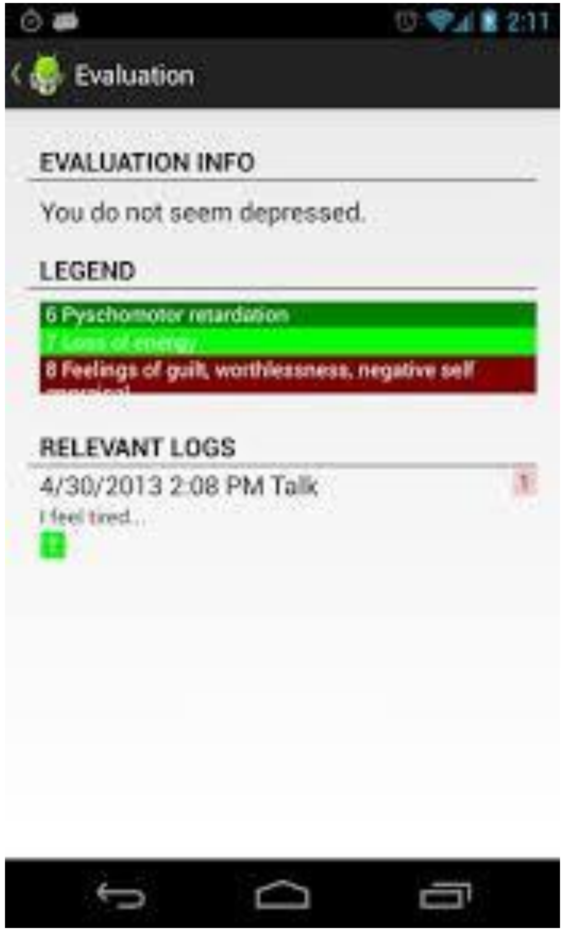

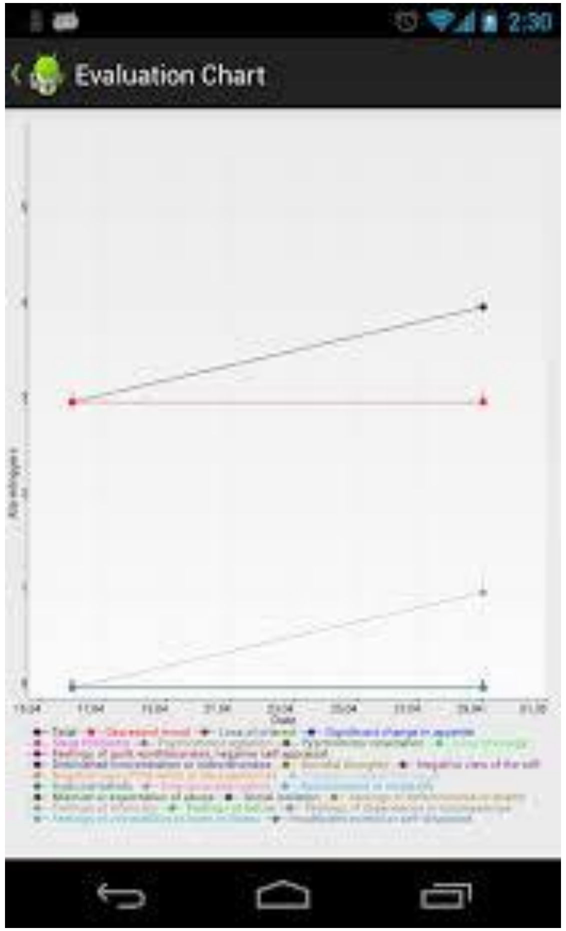

Framework

Negatively-valenced verbal behavior
Beck's Cognitive Theory

Self-focused language
Pyszczynski and Greenberg's Self-Focus Model of Depression

↘ ↙

Depressive Language

↓

***PiaP* EMA in Mobile Device**

↓

***PiaP* Text /Sentiment Analysis**
Pennebaker et al 2003; Spence, 1980, 2003

↙ ↘

↓ Depressive Symptoms
(↓"Negative" keywords, Personal pronouns)

↑ Depressive Symptoms
(↑ "Negative" keywords, Personal pronouns)

Language Use & Mental Health

- Reflection of psychological state
- Way of organizing and making sense of one's world
- Mental states and personal characteristics are reflected in the words people use in natural language (Pennebaker et al., 2003)
- Lexical leakages (Spence, Scarborough, & Ginsberg, 1978)
- Mental preoccupation with a particular theme (Spence, 1980; 2003)

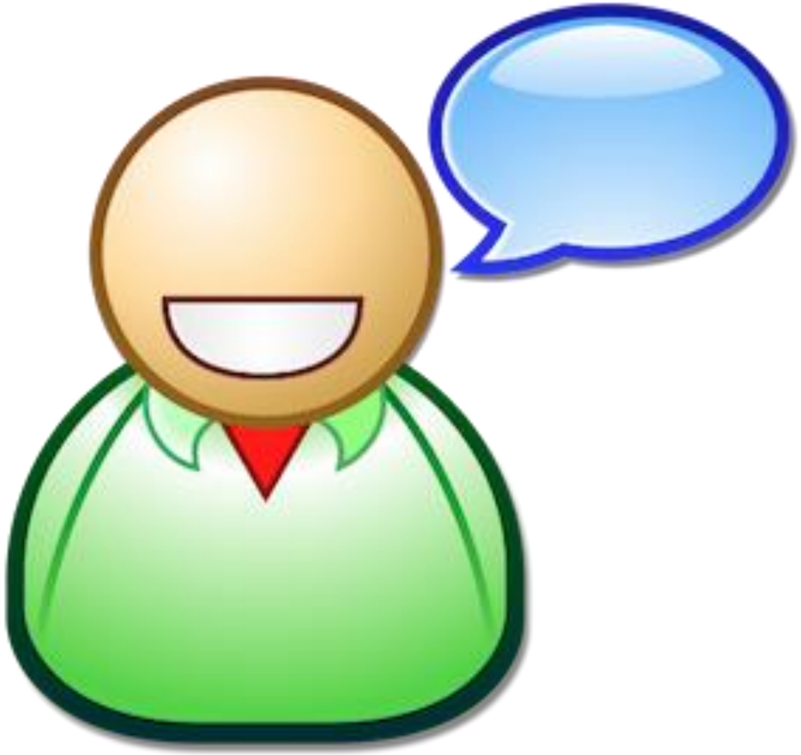

Research Objectives

1. To update and refine depression-lexicon
2. To validate depression-lexicon

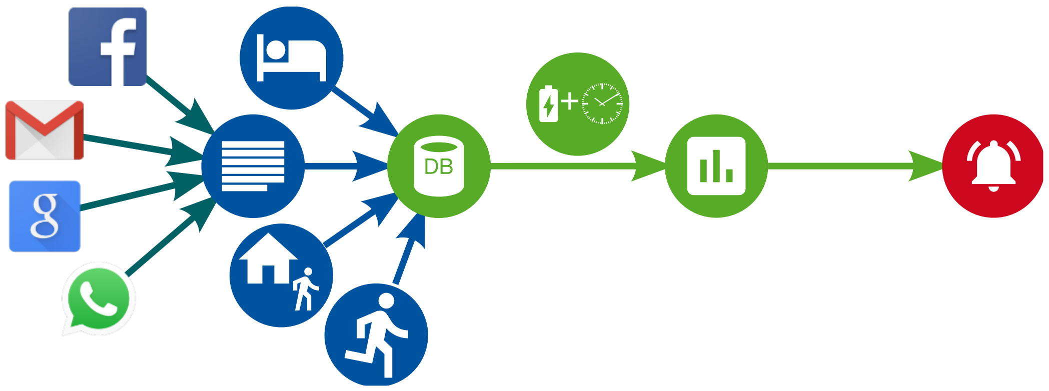

Design and Methods

Tripartite Model of Test Construction

STAGE I: Theoretical-Substantive

Generate Depression Lexicon - PiaP Draft

A. FGD on Depressive Language
B. Interview with Mental Health Professionals
C. Review of DSM IV-TR/5 Review ICD-10
D. Review of Depression Scales
E. Abbreviation of Keywords

STAGE II: Internal-Structural

Content Validity (Lawshe) **– *PiaP* Preliminary Version**

3. Item Analysis (CTT)**; Internal Consistency** (Cronbach α) **– *PiaP* Test-Run Version**

STAGE III: External-Criterion

4. Construct Validity

- Congruent (BDI-II; CESD)
- Convergent (ABS-Negative Affect)
- Divergent (ABS-Positive Affect; SWLS)

Results

Focus Group Discussion

- ❑Administered BDI-II to 811 college students
- ❑Selected 76 students (scored Mild to Severe)
- ❑7 groups, 10-11 participants each
- ❑Average 60 minutes/session
- ❑ **Focus and Themes:**
  1. Descriptions of depression – words and/or symbols typically used
  2. Revelation of depression in mobile text inputs and in social media
  3. Recognition of depression in mobile text inputs and in social media

Focus Group Discussion: Descriptions

WORDS

- 23%: “sad”, “unhappy”, “emo” and “lonely” as major descriptions (“malungkot”, hindi na masaya”)
- 12% : Having no focus (“tulala”, “lutang”, “malayo ang iniisip”) and as being disturbed or messed up (“wala sa sarili”)

SYMBOLS OR EMOTICONS

- :-(
- :-[
- =(
- ='(
- :-|
- =|
- :'-|

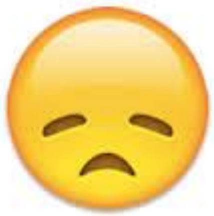

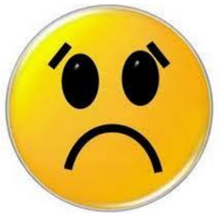

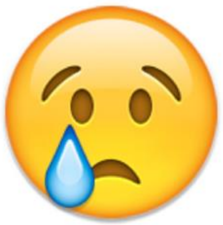

- :'-(
- :(
- T_T
- ToT
- :-/
- :-\
- :-<

Focus Group Discussion: Revelation and Recognition of symptoms

- (56%) Social Networking Sites like Twitter and Facebook
  - serve as emotional outlets
  - gives some sort of foreshadowing especially for those about to become clinically depressed
- (35%) Recognize depression in SNS posts and text messages through presence of sad words
  - personal messages
  - lyrics of sad songs or quotations

Interview with Mental Health Professionals

- 2 Clinical Psychologists; 2 Psychiatrists; 1 Facilitator of Depression Support Group
- Confirmation of found depressive keywords
- 4 Main Points:
  1. People usually tell when they are depressed
  2. Descriptions of symptoms (DSM or ICD) differ from the exact expression of depression when communicating with others
  3. Depression maybe expressed through text messages, blogs, or social networking sites
  4. An app for depression may be useful for individuals who need help

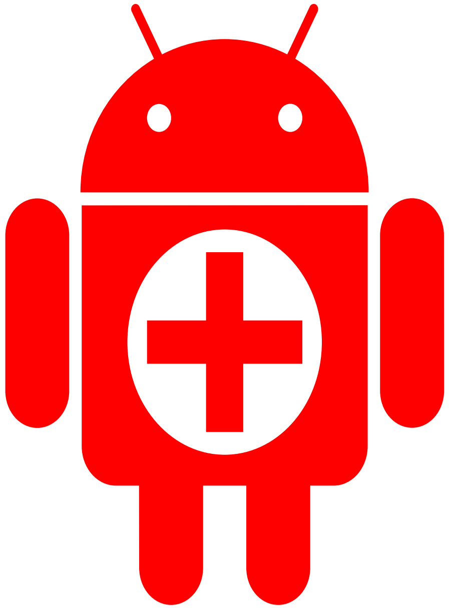

Review of 18 Depression scales

1. BDI-II (Beck Depression Inventory-II)
2. CESD (Center for Epidemiologic Studies – Depression Scale)
3. ABS (Affect Balance Scale)
4. ELMD (Experiences of Low Mood and Depression)
5. HADS (Hospital Anxiety and Depression Scale)
6. PHQ-9 (Patient Health Questionnaire-9)
7. MDI (Major Depression Inventory –ICD based)
8. IDD (Inventory to diagnose depression)
9. DASS (Depression Anxiety Scales)
10. CCI (Crandell Cognitions Inventory)
11. DAS (Dysfunctional Attitudes Scale)
12. SSSASI (Situational Self Statement and Affective State Inventory)
13. SIBI (Smith Irrational Beliefs Inventory)
14. ATQ (Automatic Thoughts Questionnaire)
15. CTI (Cognitive Triad Inventory)
16. CCL (Cognition Checklist)
17. IBI (Irrational Beliefs Inventory)
18. YSQ (Young's Early Schemas Questionnaire)

Review of 18 Depression scales

- Keyword frequency using Word Count Strategy

Category	Keywords	Frequency	Percentage
guilt and self-esteem	89	148	32.96%
mood	67	103	22.94%
interest	32	40	8.91%
anxiety	22	35	7.80%
psychomotor agitation	19	27	6.01%
fatigue	16	23	5.12%
appetite and weight	17	20	4.45%
concentration	12	17	3.79%
sleep	14	15	3.34%
suicide	9	12	2.67%
psychomotor retardation	5	8	1.78%
histrionic behavior	1	1	0.22%

Abbreviation of Keywords

- 328 conveniently selected Filipino college students in Metro Manila and Central Luzon
- Expounded to create all possible combinations of abbreviations in one string

Category	Keyword	Abbreviation 1	Abbreviation 2
Depressed Mood	Can not stop crying	Cant stp crying	Cnt stop cryin
Interest	Want to detach	Wnt 2 detach	wanna detach
Sleep	Trouble falling asleep	Trouble fallng asleep	Trble fallin aslp
Suicide	Take my life	Tke my life	Take my lyf
Guilt/Self-esteem	I am worthless	I'm wrthless	Im worthlss

PiaP Depression-Lexicon Draft

- ❑ ***13*** categories
- ❑ ***1,762*** Keywords (e.g., *Alone*)
- ❑ ***9,655*** Derivatives (e.g., *Feeling alone*)
- ❑ Abbreviations (e.g., *Feelin alone*)

Category	Keyword	Derivatives
Mood	241	1582
Interest	129	1035
Appetite & Weight	216	1357
Sleep	162	786
Motor Agitation	174	750
Motor Retardation	74	431
Fatigue	112	424
Guilt & Self Esteem	180	1000
Concentration	165	753
Suicide	90	635
Alcohol & Substance	63	315
Anxiety	112	399
Histrionic Behavior	44	188
Total	1762	9655

Content Validation

- Eight (8) Mental health professionals as expert evaluators
- Lawshe's Content Validity Index:
  - **with rejected items = 0.83**
  - **corrected CVI = 0.90**
- Retained ***1,498*** Keywords

Category	CVR
**Mood**	0.86
**Interest**	0.93
**Appetite & Weight**	0.98
**Sleep**	0.92
**Motor Agitation**	0.78
**Motor Retardation**	0.80
**Fatigue**	0.89
**Guilt & Self Esteem**	0.94
**Concentration**	0.93
**Suicide**	0.97
**Alcohol & Substance**	0.91
**Anxiety**	0.89
**Histrionic Behavior**	0.92
**Total**	**0.90**

Conclusion

- ❑Mobile technology provides an avenue for detecting depression as its serves as an emotional outlet for its users
- ❑ People reveal themselves when depressed but their exact verbal expression differ from Golden standards' depiction of depressive symptoms
- ❑ Depression scales have negatively-valenced words and statements in the first person orientation which are mostly composed of negative cognitive schemas (guilt/ low self esteem)
- ❑PiaP, a novel and culture-sensitive method, is a content valid screening tool for depressive symptoms

Ongoing Work and Future Directions

- ❑Item analysis
- ❑Construct validation
- ❑Development of depression-lexicon in German
- ❑Identification of voice features in depression
- ❑Test Plug-Ins (Mobile EEG, Mobile eye-tracker, Locator, Googlefit, Bluetooth)
- ❑Facial emotion recognition

Thank you very much for your attention.
Maraming salamat sa inyong pagkinig.
Vielen Dank für ihre Aufmerksamkeit!

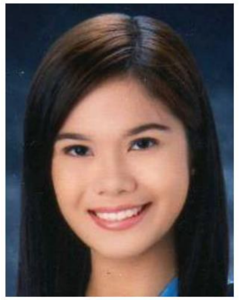

Paula Glenda Ferrer-Cheng
(ferrer_pga@yahoo.com)

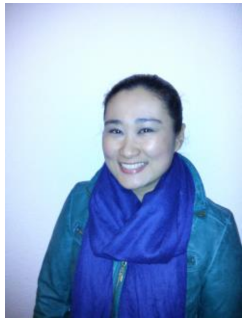

Dr Roann Munoz Ramos
(roann.ramos@gmail.com)

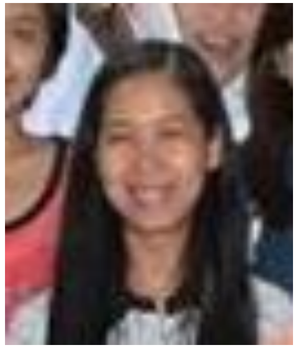

Dr Portia Lynn Quetulio-See

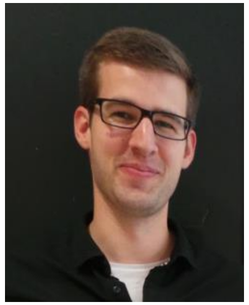

Tim Ix

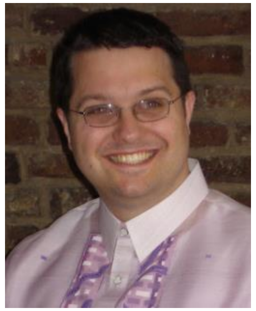

Dipl Inf Jó Ágila Link

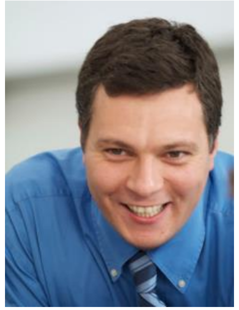

Prof Dr Klaus Wehrle

AUXILLIARY SLIDES

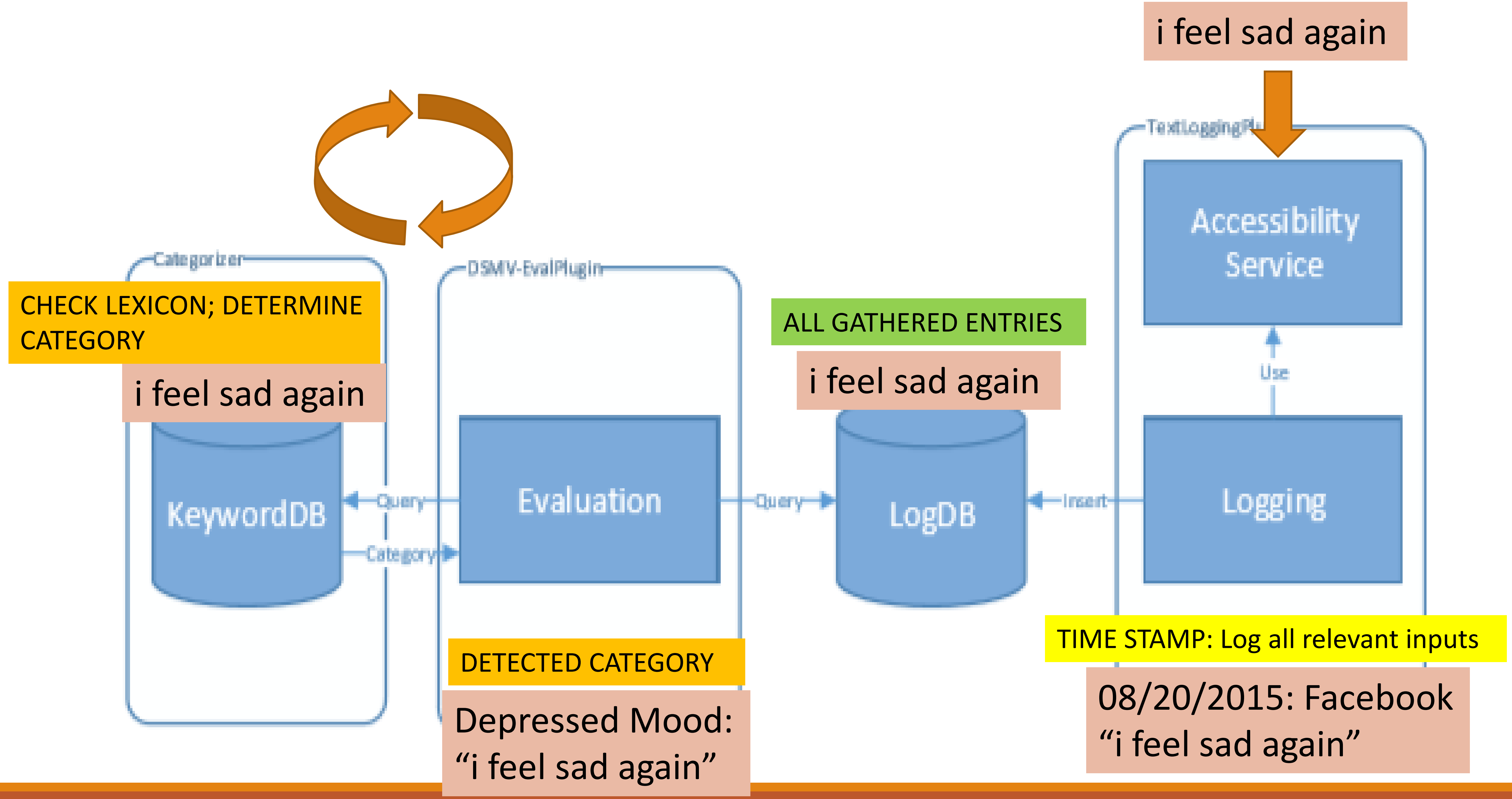
i feel sad again
Accessibility Service
Categorizer
DSMV-EvalPlugin
CHECK LEXICON; DETERMINE CATEGORY
i feel sad again
ALL GATHERED ENTRIES
i feel sad again
Use
KeywordDB
Query
Category
Evaluation
Query
LogDB
Insert
Logging
DETECTED CATEGORY
Depressed Mood: “i feel sad again”
TIME STAMP: Log all relevant inputs
08/20/2015: Facebook “i feel sad again”

Logging Component

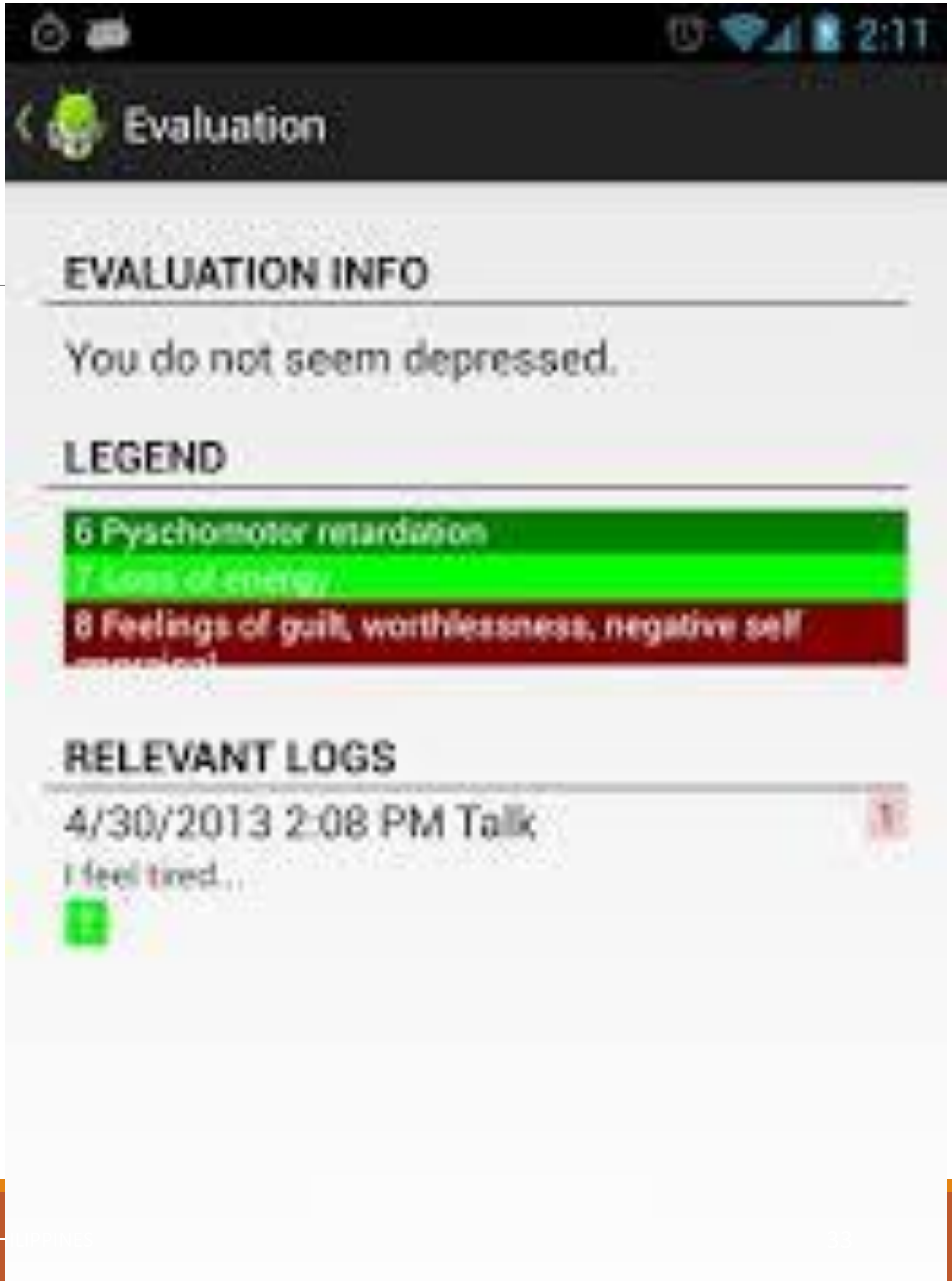

Evaluation Component

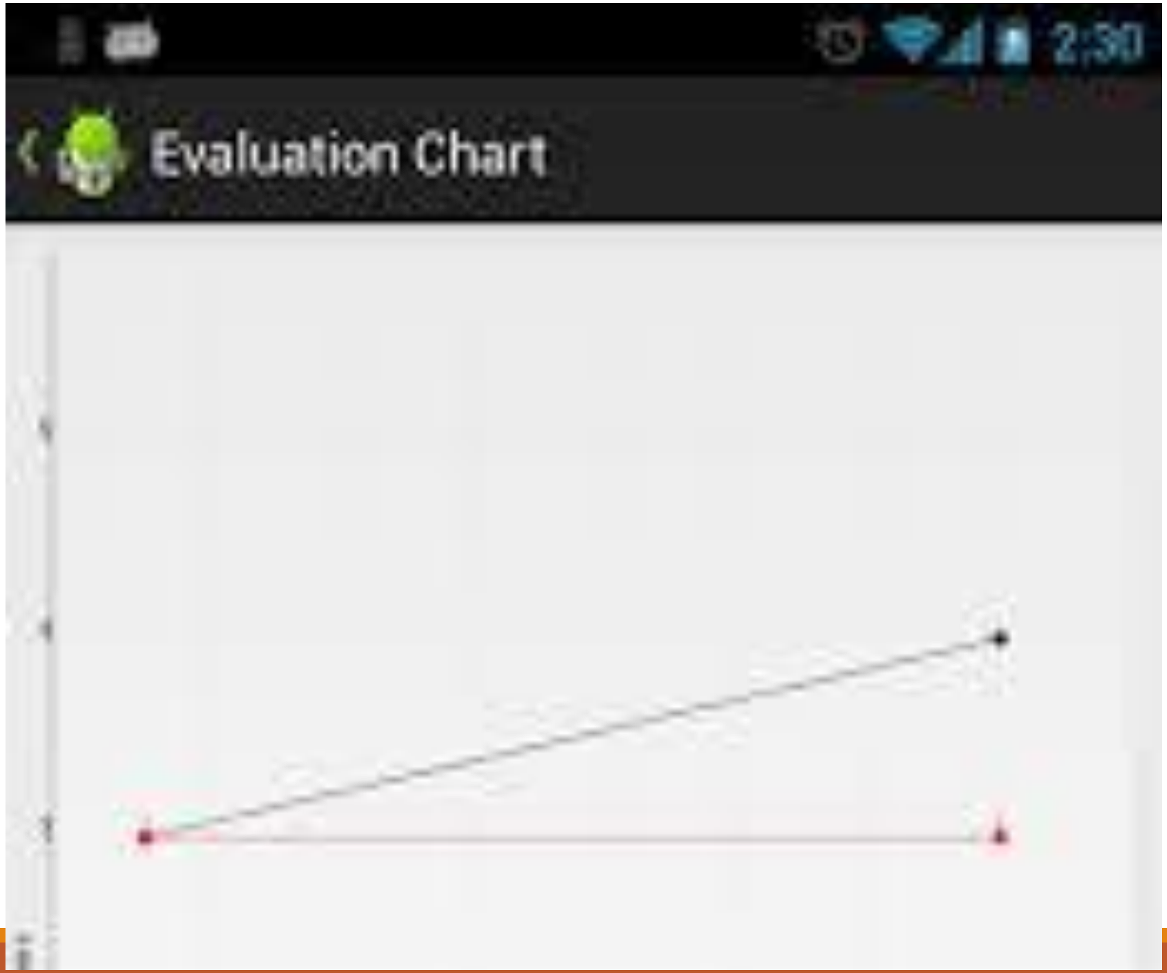

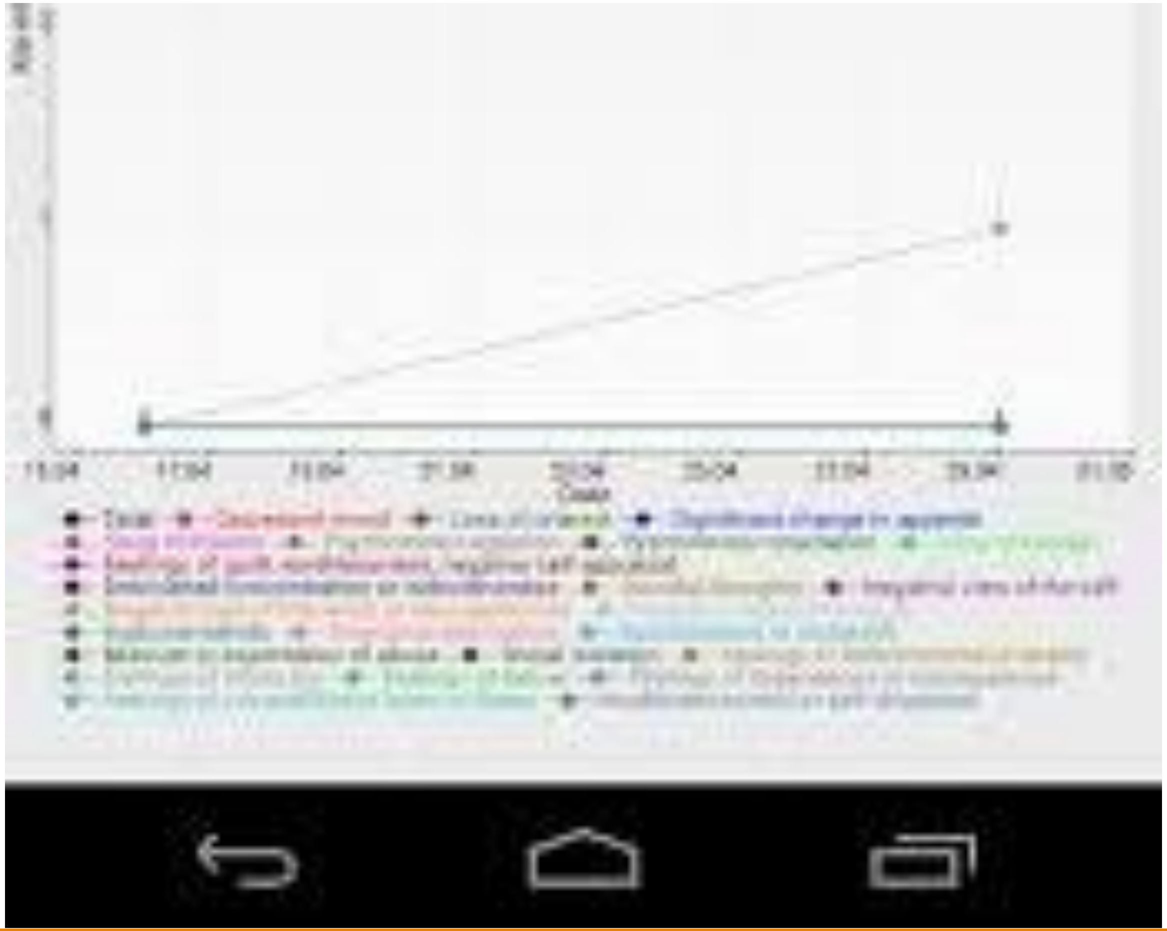

Limitations

- Text analysis technology cannot substitute the expertise of a mental health professional
- The current study involved the initial stage of the lexicon validation
- The study did not utilize crowdsourcing and relied on the responses of focus group participants who are screened with BDI-II and were found to have depressive symptoms

Tripartite Model of Test Construction

STAGE I: Theoretical Substantive

1. **Generate Depression Lexicon (Draft)**
    - FGD
    - Interview Mental Health Professionals
    - DSM & ICD-10
    - Depression Scales
    - Blogs and websites
    - Keyword Abbreviations

STAGE II: Internal-Structural

2. **Content Validity** (Lawshe)
   (Preliminary Version)

3. **Item Analysis** (CTT); **Internal Consistency** (Cronbach α)
   (Test-Run)

STAGE III: External-Criterion

4. Construct Validity
- Congruent (BDI-II; CESD)
- Convergent (ABS- Negative Affect)
- Divergent (ABS-Positive Affect; SWLS)

DSM and ICD Depressive Episode Categories

DSM IV TR/ DSM-5 Major depressive episode

1. Depressed Mood
2. Markedly diminished interest or pleasure in all or almost all activities
3. Significant (>5% body weight) weight loss or gain, or increase or decrease in appetite
4. Sleep problems
5. Psychomotor agitation or retardation
6. Fatigue or loss of energy
7. Feelings of guilt, worthlessness, negative self appraisal
8. Diminished concentration or indecisiveness
9. Recurrent thoughts of death or suicidal ideation

ICD-10 depressive episode

1. Depressed mood
2. Loss of interest and enjoyment
3. Change in appetite with corresponding weight change
4. Sleep disturbance of any type
5. Change in psychomotor activity with agitation or retardation
6. Reduced energy leading to increased fatigability and diminished activity
7. Unreasonable feelings of self-reproach or excessive and inappropriate guilt
8. Loss of self-esteem and self-confidence
9. Reduced concentration and attention
10. Recurrent thoughts of death or suicidal ideation or any suicidal behavior
11. Anxiety
12. Excessive consumption of alcohol
13. Histrionic Behavior

Review of Depression scales

1. BDI-II (Beck Depression Inventory-II)
2. CESD (Center for Epidemiologic Studies – Depression Scale)
3. ABS (Affect Balance Scale)
4. ELMD (Experiences of Low Mood and Depression)
5. HADS (Hospital Anxiety and Depression Scale)
6. PHQ-9 (Patient Health Questionnaire-9)
7. MDI (Major Depression Inventory –ICD based)
8. IDD (Inventory to diagnose depression)
9. DASS (Depression Anxiety Scales)
10. CCI (Crandell Cognitions Inventory)
11. DAS (Dysfunctional Attitudes Scale)
12. SSSASI (Situational Self Statement and Affective State Inventory)
13. SIBI (Smith Irrational Beliefs Inventory)
14. ATQ (Automatic Thoughts Questionnaire)
15. CTI (Cognitive Triad Inventory)
16. CCL (Cognition Checklist)
17. IBI (Irrational Beliefs Inventory)
18. YSQ (Young's Early Schemas Questionnaire)
