# Supplementary material for: Psychologist in a Pocket: Lexicon Development and Content Validation of a Mobile-Based App for Depression Screening
Source: JMIR Mhealth Uhealth. 2016 Jul 20;4(3):e88. doi: 10.2196/mhealth.5284 (PMC4972990; doi:10.2196/mhealth.5284)
Supplement: Multimedia Appendix 2 [file mhealth_v4i3e88_app2.pdf]

+

HOW DO I SAY "SAD"?

Building a depression lexicon for *Psychologist in a Pocket*

ROANN MUNOZ RAMOS, PhD
mHealth Division, RWTH Aachen University Hospital
Aachen, Germany

InPACT International Psychological Applications Conference & Trends
Lisbon, Portugal
30 April – 02 May 2016

UNIKLINIK RWTHAACHEN

Language & Depression

- Language:
  - Reflects one's thoughts
  - (Spence, Scarborough, & Ginsberg, 1978) Lexical leakages
  - Indicator of mental health
  - As a diagnostic approach

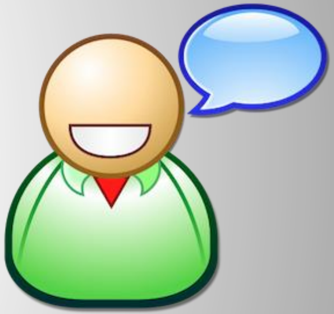

- In depression:
  - (Cognitive Behavioral Theory) Role of negative schemas in views of self, world, future
  - (W Weintraub) 1st person pronouns
  - (Self-focus Model) Attention to self triggers negative self schema

Psychologist in a Pocket

- (Bitsch et al., 2015) Android smartphone application for depression screening via text analysis (electronic data)
- Logged inputs compared against a language-specific depression lexicon
- Ecological Momentary Analysis (EMA): Detects fleeting moods, gathers data passively, allows for privacy
- As an adjunct in depression screening and monitoring

PiaP Lexicon Development

- Lexicon development: Top-down (deductive/gold-standard) and bottom-up (inductive) processes
- Use of established classification systems: DSM 5 & ICD 10
  - PiaP 13 symptoms categories (e.g., Depressed Mood, Sleep Problems)
- Review of 18 depression tests
- Focus group discussions: n: 76 (10-11 participants/session); (Mean age: 17.28 yrs.; 46 females)
  - Linguistic expressions of depression
- Consultations with mental health experts
- Survey on spelling variations/text writing culture: N: 328 university students

UNIKLINIK RWTHAACHEN

PiaP Lexicon Development: Depression Tests

- 18 Tests (e.g., BDI-II, CES-D Scale, PHQ-9)
- Top 3 keywords: Guilt & lowered self-esteem, depressed mood, decreased interest
- 1st person POV
- Associated symptoms not covered

Table 1. Keyword Frequency from Depression Tests

Category	Keyword	Frequency	%	Cumulative %
Guilt & Self-esteem	89	148	32.96	32.96
Mood	67	103	22.94	55.90
Interest	32	40	8.91	64.81
Anxiety	22	35	7.80	72.61
Psychomotor agitation	19	27	6.01	78.62
Fatigue	16	23	5.12	83.74
Appetite & Weight	17	20	4.45	88.20
Concentration	12	17	3.79	91.98
Sleep	14	15	3.34	95.32
Suicide	9	12	2.67	98.00
Psychomotor retardation	5	8	1.78	99.78
Histrionic behavior	1	1	0.22	100.00

PiaP Lexicon Development: FGD & Survey

- 78 responses: Sad, lonely, unhappy
- 67% of 66 responses reveal depression online
- 35% of 50 responses indicate that they can recognize depression from SNS posts and text messages
- Textolog and Taglish: Word “shortening” and mixing Filipino/Tagalog and English words
- Emojis and emoticons

Table 2. FGD Descriptions of Depression

Responses	%	Cumulative %
Sad; Lonely; Unhappy	27	27
No focus; Disturbed	14	41
Isolation; Lack of interest; Low interaction	9	50
Sleep problems	9	59
Hopelessness; Loss of meaning in life	8	67
Fatigue; Stressed	6	73
Pessimism	5	78
Uneasiness; Instability	5	83
Moody	4	87
Emotional	3	90
Eating problems	3	92
Low self-esteem	3	95
Suicidal	1	96
Anxiety	1	97
Have no emotional support	1	99
Pretending to be happy	1	100

Table 3. Sample Spelling Variations

Category	Keyword	Variation 1	Variation 2
Depressed Mood	Can not stop crying	Cant stp crying	Cnt stop cryin
Interest	Want to detach	Wnt 2 detach	wanna detach
Sleep	Trouble falling asleep	Trouble fallng asleep	Trble fallin aslp
Suicide	Take my life	Tke my life	Take my lyf
Guilt/Self-esteem	I am worthless	I'm wrthless	Im worthlss

PiaP Lexicon Development

- **Bilingual lexicon**
- **Total Keywords: 835,286**

  Main keywords + derivatives

  Spelling variations

- **61 First person pronouns + spelling variations**
- **187 emoticons and emojis**

Research Directions & Conclusions

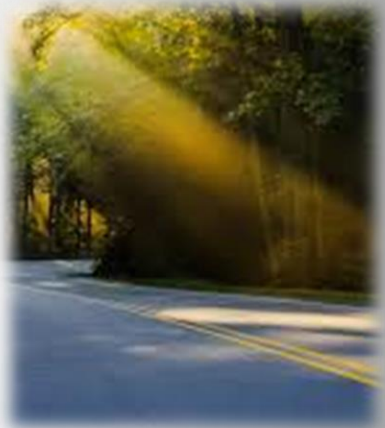

- Research directions:
  - Initial validation with university students from Manila, PH
  - German lexicon
  - Behavioral indicators (e.g., location, sleep)
  - Inclusion of CBT
- To conclude:
  - Immediate detection of depression symptoms maybe improved
  - Discussions for scientific, ethical and legal dimensions of using mhealth applications
